# Fabrication and *In Vitro* Evaluation of 3D Printed Porous Polyetherimide Scaffolds for Bone Tissue Engineering

**DOI:** 10.1155/2019/2076138

**Published:** 2019-11-11

**Authors:** Xiongfeng Tang, Yanguo Qin, Xinyu Xu, Deming Guo, Wenli Ye, Wenzheng Wu, Ruiyan Li

**Affiliations:** ^1^Department of Orthopedics, The Second Hospital of Jilin University, Jilin University, Changchun 130041, China; ^2^Department of Mechanical Manufacturing and Automation, School of Mechanical Science and Engineering of Jilin University, Changchun 130025, China

## Abstract

For bone tissue engineering, the porous scaffold should provide a biocompatible environment for cell adhesion, proliferation, and differentiation and match the mechanical properties of native bone tissue. In this work, we fabricated porous polyetherimide (PEI) scaffolds using a three-dimensional (3D) printing system, and the pore size was set as 800 *μ*m. The morphology of 3D PEI scaffolds was characterized by the scanning electron microscope. To investigate the mechanical properties of the 3D PEI scaffold, the compressive mechanical test was performed via an electronic universal testing system. For the *in vitro* cell experiment, bone marrow stromal cells (BMSCs) were cultured on the surface of the 3D PEI scaffold and PEI slice, and cytotoxicity, cell adhesion, and cell proliferation were detected to verify their biocompatibility. Besides, the alkaline phosphatase staining and Alizarin Red staining were performed on the BMSCs of different samples to evaluate the osteogenic differentiation. Through these studies, we found that the 3D PEI scaffold showed an interconnected porous structure, which was consistent with the design. The elastic modulus of the 3D PEI scaffold (941.33 ± 65.26 MPa) falls in the range of modulus for the native cancellous bone. Moreover, the cell proliferation and morphology on the 3D PEI scaffold were better than those on the PEI slice, which revealed that the porous scaffold has good biocompatibility and that no toxic substances were produced during the progress of high-temperature 3D printing. The osteogenic differentiation level of the 3D PEI scaffold and PEI slice was equal and ordinary. All of these results suggest the 3D printed PEI scaffold would be a potential strategy for bone tissue engineering.

## 1. Introduction

Bone tissue engineering is a promising approach to heal the bone defect caused by fractures, infection, or tumor [1]. A key factor in bone tissue engineering for bone regeneration is the scaffold that plays the role of a template for cell adhesion, cell proliferation, and formation of the bone-extracellular matrix to provide structural support to the newly formed bone. The scaffolds should mimic the bone structure and function in order to optimize the integration between the scaffold and the surrounding tissue [2]. A porous structure scaffold can improve the mechanical properties to match the bone tissue [3, 4] and provide a cell-friendly structure microenvironment for the growth of osteoblasts and tissues. Furthermore, the inner surface of the porous scaffold is much larger than the solid implant, which makes it possible to carry drugs and growth factors to enhance bone healing [5]. Therefore, the porous scaffold is one of the hotspots for bone tissue engineering. Although a porous scaffold could be fabricated by traditional methods, only a randomly organized porous structure can be achievable. 3D printing, a scaffold fabrication method, can fabricate suitable customized porous scaffolds for bone tissue engineering research and orthopedics surgery [6, 7]. As the development of 3D printing technologies, such as electron beam melting (EBM), selective laser sintering (SLS), fused deposition modeling (FDM), and stereolithography (SLA), the fabrication of scaffolds with the complex 3D structure becomes relatively easy [8, 9].

Metal and polymer are the most commonly used materials for the fabrication of 3D printed bone tissue scaffolds [10]. Among the metal materials, titanium alloy is most widely used in the research and clinical application in orthopedics [2]. However, the elastic modulus of titanium alloy is about 110 GPa, which is still much higher than that of the cortical bone (about 10–30 GPa) [11, 12]. Polymer materials, especially the degradable polymers, such as polylactide (PLA), polycaprolactone (PCL), and poly(lactic-co-glycolic acid) (PLGA), are often used as implants of non-load-bearing parts for bone defect repair because of their excellent biocompatibility and low toxicity [13, 14]. Generally, the degradable polymers are much easier to prepare compared with the metal, but their weak load-bearing capacity limits their application. Special engineering plastics combine the advantages of both metal and degradable polymers. Polyetheretherketone (PEEK) and polyetherimide (PEI), as the typical representatives, have good biocompatibility and corrosion resistance, and they have similar elastic modulus to the trabecular bone compared to titanium [15, 16]. Moreover, the melting point of them is much lower than that of titanium alloys, making them relatively easy to be 3D printed.

The mechanical properties of pure PEI are close to those of pure PEEK, and strength of both of them can be enhanced to the level of the cortical bone via doping other materials, such as carbon or glass fiber [17–19]. The usable temperature of PEI is −20° to 335°F, which is lower than that of PEEK, but it does not need to work in extreme temperatures as a bone tissue scaffold. As an advantage, the cost of PEI is lower than that of PEEK, and it has similarity to the physiological structure of the bone in charge transfer [20–22]. Meanwhile, for its biocompatibility, PEI has been proved as a potential membrane production material for biohybrid organ systems and hemodialysis [23, 24]. At present, there are a large number of researches about 3D printed PEEK scaffolds in orthopedics. However, there was no study that reported on PEI biocompatible scaffolds fabricated via 3D printing. This work is the first report to investigate the possibility of 3D printed porous PEI used as a biocompatible scaffold in bone tissue engineering.

Previous studies show that pore size had significant influence on the growth of cells in the scaffolds. The scaffolds with mean pore sizes ranging from 300 *μ*m to 1000 *μ*m were deemed optimal for bone tissue engineering [25, 26]. In this study, we fabricated the PEI scaffold with interconnected micropores via the FDM 3D printer, and the pore size was set as 800 *μ*m. For analysis, its feasibility as a bone tissue scaffold and the elastic modulus, cell adhesion, cytotoxicity, cell proliferation, and osteogenic differentiation of the 3D printed scaffold were evaluated, and the results suggested that 3D printed PEI is a promising candidate as a bone tissue scaffold.

## 2. Materials and Methods

### 2.1. Fabrication of Samples

The PEI rods and grains were purchased from Sigma-Aldrich in USA. A homemade 3D printer (provided by the School of Mechanical Science and Engineering of Jilin University) was used to fabricate the 3D PEI scaffolds (3D PEI). Firstly, the PEI grains were melted and extruded into filaments (Φ = 1.75 mm) (SJZS-10 twin-screw extruder, Wuhan Running Company). Secondly, the filaments were dried by keeping at a constant temperature of 60°C with desiccants for 24 hours. Then, the dried PEI filaments were added into the heated nozzle 3D printer, and the temperature was kept at 365°C. According to the options of the 3D printer and slicing data of the 3D model, the PEI filament was melted and extruded into the designed shape (length*∗*width*∗*height=20 mm*∗*12 mm*∗*12mm), and the thickness of each layer was 0.8 mm, which had ordered arrays and uniform mesh.

### 2.2. Physical Characterizations

The compressive mechanical test was performed on the 3D PEI scaffolds (20 ∗ 12 ∗ 12 mm) and PEI rods (12.7 mm ∗ 10 mm) via the electronic universal testing system (Instron 5869, USA) with 50 kN load cells and the crosshead speed set as 1 mm/min. The compressive strength, compressive modulus, and stress-strain curve were obtained from the load recorded. The structure and the surface of the 3D PEI scaffold were observed via FE-SEM (XL-30 ESEM FEG Scanning Electron Microscope, FEI Company). The samples were sputtered with Au before SEM observation.

### 2.3. Cell Viability and Cytotoxicity

The rabbit bone marrow stromal cells were obtained from the 28-day fetal Japanese White rabbit (College of Veterinary Medicine, Jilin University, China). All the long limb bones were dissected from the attached soft tissues, and then BMSCs were obtained through flushing the bone marrow cavities with basic culture medium according to the previous description [27], and cells between the third and the fifth passage were used in the following *in vitro* experiments. The PEI rods were cut into PEI slices with a size of Φ 12.7 mm ∗ 1.5 mm, while the 3D PEI scaffolds were cut into 10 mm ∗ 12 mm ∗ 2 mm for cell culture. All kinds of samples were ultrasonically cleaned in deionized water and sterilized with a high-pressure steamer at 120°C for 70 min. The samples were kept sterile for subsequent experiments after drying. PEI slice, 3D PEI scaffold, and titanium alloy (Ti6Al4V) groups were, respectively, soaked in a centrifuge tube with basic culture medium (Dulbecco's modified Eagle's medium (DMEM), low glucose) for 24 hours at 37°C and then supplemented with 1% penicillin-streptomycin solution and 10% fetal bovine serum to make the leaching solution for cytotoxicity. Ten samples of each group were used to prepare the leaching solution. Ten PEI samples and ten 3D PEI samples had the same weight. Cells were cultured in a 24-well plate and a 96-well plate at a density of 1 × 10^4^ cells/ml for 24 h, and then the medium was replaced by the leaching solution. After culturing for another 24 h, the live/dead cell staining was performed on the cells in 24-well plates using LIVE/DEAD Cell Viability Assays (Invitrogen, Life Technologies, Carlsbad, CA, USA). The dyes were component A (calcein AM, 2 mM) and component B (PI solution, 1.5 mM). The experiment was performed as described in the manual. The samples were imaged using a scanning fluorescence microscope (Olympus BX51TF, Japan). Cells in 96-well plates were cultured for another 48 h. The plate with the normal culture group and the Ti6Al4V group was set as the control. Then, the cytotoxicity was measured using Cell Counting Kit-8 (CCK-8, Dojindo, Japan) at 450 nm using a microplate reader (Varioskan Flash, Thermo Scientific).

### 2.4. Cell Adhesion and Morphology

In order to investigate the adhesion of BMSCs in different groups, cells were seeded in two different samples of each group at a density of 5 × 10^4^ cells/ml. After 12 h of incubation, the samples were transferred to a new plate and smoothly washed 3 times with PBS and then fixed with 4% paraformaldehyde for 30 min at 4°C. Fixed samples were washed again with PBS for 2 min. The cell nuclei were stained with 4ʹ,6-diamidino-2-phenylindole (DAPI, Sigma-Aldrich, USA) for 5 min and were observed by a scanning fluorescence microscope (Olympus BX51TF, Japan). In order to directly observe the morphology of the cells on the scaffold, cells were cultured on different samples at a density of 1 × 10^4^ cells/ml for 3 days. Then, the samples with cells were rinsed 3 times with PBS for 2 min, fixed with 2.5% v/v glutaraldehyde at 4°C for 8 h, and dehydrated through an ethanol series. The samples were sputtered with Au before SEM observation (XL-30 ESEM FEG Scanning Electron Microscope, FEI Company).

### 2.5. Cell Proliferation Assay

For the cell proliferation assay, which is performed twice, cells were seeded on the PEI slice and 3D PEI scaffold, at a density of 1 × 10^4^ cells/ml in 24-well plates, and three samples in each group were treated as the parallel control. The CCK-8 reagent was used to measure the number of cells after culture for 1, 4, and 7 days. A mixed solution of CCK-8 and DMEM in a ratio of 10 : 100 was added into wells and incubated at 37°C for 2 h. The number of viable cells at each time point was measured via the absorbance of optical densities (OD) at 450 nm using a microplate reader (Varioskan Flash, Thermo Scientific).

### 2.6. Osteogenic Differentiation

For the evaluation of the osteogenic differentiation, the BMSCs were seeded on PEI and 3D PEI samples at a density of 1 × 10^4^ cells/ml in 24-well culture plates with DMEM. Then, the medium was replaced by the osteogenic medium (basic culture medium containing 50 mg/L ascorbic acid, 10^−8^ M dexamethasone, and 10 mM *β*-glycerol phosphate; Cyagen, China).

After osteogenic induction for 7 days, the BCIP/NBT Alkaline Phosphatase Color Development Kit (Beyotime, China) was employed to quantify the alkaline phosphatase (ALP) secretion by staining the samples according to the manufacturer's instructions. Images were acquired by a zoom stereo microscope (Canon, Japan). After induction for 14 days, the samples and cells were fixed with 4% paraformaldehyde for 20 min at 4°C. Fixed samples were washed twice with PBS for 3 min, and the Alizarin Red kit was added into the well with samples. After the samples were stained with the Alizarin Red kit for 40 min in dark, the samples were washed again with PBS, and the images were observed via a zoom stereo microscope (Canon, Japan).

### 2.7. Statistical Analysis

Three to five samples per group per time point were used in different experiments, while 10 samples of each group were used to prepare the leaching solution. The results are presented as mean ± standard deviation (SD) for each group. Statistical differences were analyzed using an analysis of *t*-test in this study. The *P* value <0.05 was considered significant.

## 3. Results and Discussion

**Figure 1:**
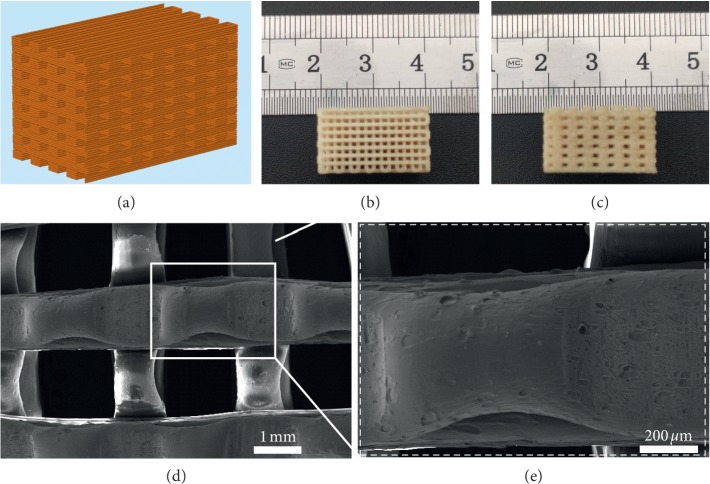
Visualization of the 3D PEI scaffold: design diagram (a); top view (b) and cross-sectional view (c) photos of the 3D PEI scaffold; SEM images of the structure and surface of the scaffold (d, e).

Figure 1 shows the design diagram (Figure 1(a)), top view (Figure 1(b)) and cross-sectional view (Figure 1(c)) photos, and SEM images (Figures 1(d) and 1(e)) for the 3D PEI scaffold. It can be seen that the sample presented a regular layer-by-layer structure with interconnected micropores, which is consistent with the design diagram. As shown in the low-magnification SEM image (Figure 1(d)), the PEI filaments were cross-stacked and formed a microporous structure. As shown in the high-magnification image (Figure 1)(e), the surface of the filament is smooth with few small pits, which may be caused by the breakdown of tiny bubbles generated during high-temperature printing. This result suggests that polyetherimide can be well fabricated into the porous scaffold via fused deposition modeling. It is well known that the microporous tissue engineering scaffold facilitates the transport of water, nutrients, and oxygen, and it also gives much more space for tissue growth into the scaffold [28–30]. In this work, the pore size was set as about 800 *μ*m, which is in the range of the optimal pore size for porous implants [26].

**Figure 2:**
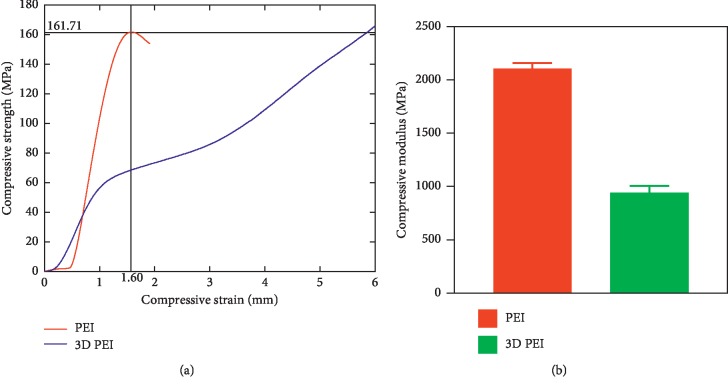
Stress-strain curves (a) and compressive modulus (b) of the 3D PEI scaffold and PEI slice.

Figure 2 shows the stress-strain curve for PEI and 3D PEI groups. The maximum stress of PEI was 161.0 ± 1.0 MPa, and the elastic modulus of the 3D PEI scaffold was 941.33 ± 65.26 MPa, which was lower than that of PEI (2106.67 ± 51.32 MPa). The range of elastic modulus for the cancellous bone is about 50–3000 MPa [31–33], and therefore, the mechanical behavior of the 3D PEI scaffold meets the requirement of the implant for filling cancellous bone defects.

**Figure 3:**
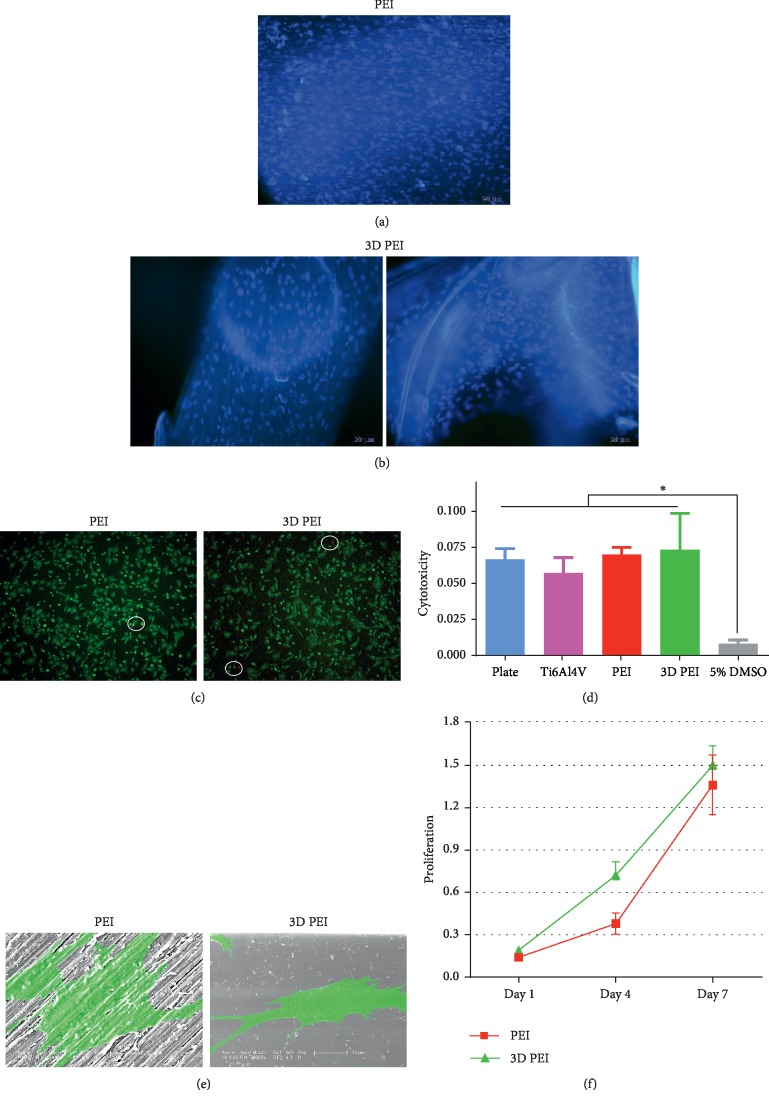
Results of biocompatibility analysis for the 3D PEI scaffold and PEI slice. (a, b) Fluorescence images of nuclei of different samples. Scale: 200 *μ*m. (c) Live/dead cell staining. Scale: 100 *μ*m. (d) Cytotoxicity test. (e) SEM images of cell morphology. Scale: 20 *μ*m. (f) Cell proliferation. ^∗^Statistically significant at *p* < 0.05 vs. the control.

As a bone tissue scaffold, biocompatibility is the initial requirement. Hence, the staining of nuclei, live/dead cell staining, cytotoxicity test, cell morphology, and cell proliferation were performed, and the results are shown in Figure 3.  Figure 3(a) shows that lots of cells adhered on the 2D surface of the PEI group, while Figure 3(b) clearly displays the cell adhesion on the struts and corners of the porous 3D PEI. In Figure 3(c), live cells are stained green and dead cells are stained red, in which both 3D PEI scaffold and PEI slice groups show mainly living cells and rarely dead cells.  Figure 3(d) shows the result of the cytotoxicity test, in which the number of cells in the 5% DMSO group is significantly lower than that in the other four groups, and there are statistical differences. There is no statistical difference between PEI, 3D PEI, and Ti6Al4V groups and plate group, whereas the performances of PEI and 3D PEI are better than that of Ti6Al4V. The result of 3D PEI is comparable to that of plate group in this cytotoxicity test, which means that they are basically nontoxic.  Figure 3(e) shows the false-color SEM images of cells on the surface of PEI and 3D PEI scaffolds. Cells in the 3D PEI group show more filopodia compared with those in the PEI group. In order to analyze the effect of the 3D PEI scaffold on cell proliferation, the number of cells at 1, 4, and 7 days was detected via the CCK-8 kit. As shown in Figure 3(f), cells in both PEI group and 3D PEI group showed an increasing trend, while the number of cells in the 3D PEI group was higher than that in the PEI group at all three time points, and the difference was statistically significant after culture for 4 days. These results suggest that both PEI and 3D PEI scaffolds have good biocompatibility and that no toxic substances were produced during high-temperature 3D printing. Good cell adhesion is a prerequisite for cell proliferation and migration. The staining of nuclei and the SEM images of cell morphology demonstrate that porous 3D PEI provided a good environment conducive to cell adhesion [34]. Furthermore, the proliferation curve indicates that the 3D PEI scaffold is more conducive to cell growth compared to the PEI slice group because of the larger inner surface of the porous structure [35]. As mentioned above, PEI has been proved as a potential membrane production material which has been applied into biohybrid organ systems and hemodialysis. Meanwhile, PEI also has been applied as bioreactors in dental and oral tissue engineering [36]. All these clinical applications indicate that both PEI and its membranes have excellent biocompatibility. Compared with the PEI membrane on other material scaffolds, 3D PEI can serve as a template for cell ingrowth and the formation of new bone tissue [37].

**Figure 4:**
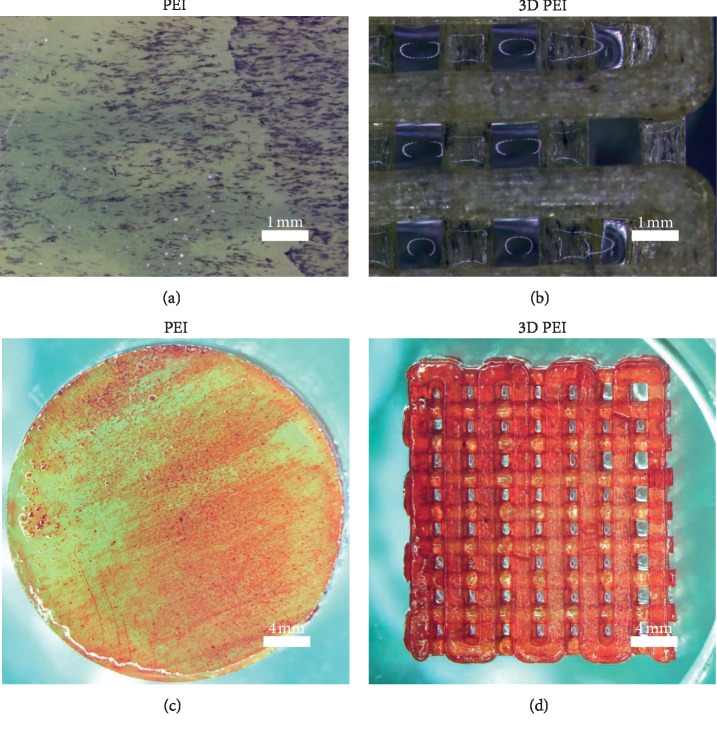
ALP staining (a, b) and Alizarin Red staining (c, d) images of the 3D PEI scaffold and PEI slice.

The secretion of ALP is an important indicator for evaluating the osteogenic differentiation of biomaterials. After osteogenic induction for 7 days, cells in 3D PEI scaffold and PEI groups were examined using ALP staining. As shown in Figures 4(a) and 4(b), both PEI and 3D PEI scaffold groups display lots of blue-purple spots (ALP-positive areas). The extracellular matrix mineralization is another important indicator for evaluating the osteogenic differentiation, and the mineralized nodules could be observed by Alizarin Red staining.  Figure 4(b) shows the Alizarin Red staining of different samples after osteogenic induction for 14 days. It can be seen that the red-stained areas on the 3D PEI scaffold were slightly deeper than those on the PEI group, but the number of calcium nodules in both groups was similar. To sum up, the osteogenic differentiation level of the 3D PEI scaffold and PEI slice was equal and ordinary. In future studies, we would enhance their osteogenic differentiation through surface treatment.

## 4. Conclusions

In this work, we have designed and fabricated 3D scaffolds using polyetherimide as a raw material via a homemade 3D printer. The 3D PEI scaffold showed an interconnected porous structure, and its elastic modulus was 941.33 ± 65.26 MPa, which falls in the range of modulus for the native cancellous bone. The *in vitro* cell experiment demonstrates that the 3D PEI scaffold has good biocompatibility, and the porous structure provides a more suitable environment for cell proliferation compared with the PEI slice. However, the osteogenic differentiation level of the 3D PEI scaffold and PEI was ordinary. According to the biocompatibility and mechanical properties of the 3D PEI scaffold, we can consider it a potential strategy for bone tissue engineering, but the bioactivity of the surface should be improved in future studies.

## Data Availability

The data used to support the findings of this study are available from the corresponding author upon request.
